# Long noncoding RNA ROR promotes breast cancer by regulating the TGF-β pathway

**DOI:** 10.1186/s12935-018-0638-4

**Published:** 2018-09-18

**Authors:** LingLi Hou, Jiancheng Tu, Fangxiong Cheng, Hongwei Yang, Fei Yu, Minghua Wang, Jiubo Liu, Jinbo Fan, Guojun Zhou

**Affiliations:** 1 Department & Program of Clinical Laboratory, Zhongnan Hospital, Wuhan University, No. 169 Donghu Road, Wuhan, 430071 Hubei People’s Republic of China; 2 Department of Blood Transfusion, Taihe Hospital Affiliated to Hubei University of Medicine, Shiyan, 442000 Hubei People’s Republic of China; 3 Department of Clinical Laboratory, Puai Hospital, Tongji Medical College, Huazhong University of Science and Technology, No. 473 Hanzheng Street, Wuhan, 430033 Hubei People’s Republic of China; 4 Department of Clinical Laboratory, Taihe Hospital, Hubei University of Medicine, Shiyan, 442000 Hubei People’s Republic of China; 5 Department of Clinical Laboratory, People’s Hospital of Yunxi County of Hubei Province, Yunxi, 442600 Hubei People’s Republic of China; 6 Department of Breast and Thyroid Surgery, Taihe Hospital, Hubei University of Medicine, Shiyan, 442000 Hubei People’s Republic of China

**Keywords:** Long noncoding RNA, ROR, TGF-β, Breast cancer

## Abstract

**Background:**

Breast cancer is the leading cause of oncological mortality among women. Efficient detection of cancer cells in an early stage and potent therapeutic agents targeting metastatic tumors are highly needed to improve survival rates. Emerging evidence indicates that lncRNAs (long noncoding RNAs) are critical regulators of fundamental cellular processes in a variety of tumors including breast cancer. The functional details of these regulatory elements, however, remain largely unexplored.

**Methods:**

In this study, lncRNA ROR (linc-ROR) was examined by real-time PCR in different breast cancer cell lines and breast tumor tissues/non-tumor tissues were collected from both breast cancer patients and healthy controls. Linc-ROR was knockdown in breast cancer cell lines and the effects on cell proliferation, migration and invasion were tested both in vitro and in vivo tumor model. Effects of linc-ROR knockdown on TGF-β signaling pathway were investigated by Western blot.

**Results:**

Our studies have suggested that linc-ROR, a critical factor for embryonic stem cell maintenance, probably acts as an oncogenic factor in breast cancer cells, causing poor prognostic outcomes. Overexpression of linc-ROR seems to be responsible for promoting proliferation and invasion of cancer cells as well as tumor growth in nude mice. The regulatory action of linc-ROR can affect the activity of the TGF-β signaling pathway, which has been proven critical for mammary development and breast cancer.

**Conclusions:**

The results have highlighted the potential importance of linc-ROR in the progression of advanced breast cancer, and thus will stimulate efforts in the development of novel diagnostic and therapeutic strategies.

## Background

Breast cancer is the most common cancer and the leading cause of oncologic mortality for women worldwide [1]. The primary risk factors of this disease include age, high hormone level, race, economic status, and iodine deficiency in diet [2]. Early diagnosis is key to successful treatment of breast cancer. Diagnostic procedures often include clinical examination, mammography, ultrasound, magnetic resonance imaging and biopsy. Standard treatment often requires complete tissue removal, chemotherapy, radiotherapy, and hormone therapy [3]. Many studies aimed to demonstrate the gene expression profiles of breast cancer have led to the recognition that the disease is highly heterogeneous and newly discovered molecular markers have allowed categorization of the disease into several biological subtypes, ultimately enabling the optimization of clinical practices in many aspects, such as prediction of prognosis and treatment responses [4–6]. Despite the overall advancement in the field, metastatic relapse of the disease at the advanced stage remains a major problem.

The non-protein-coding regions in human genome have been well recognized as important regulatory and functional units, most of which encode long non-coding RNAs (lncRNAs) [7]. Like, protein-coding mRNA, many lncRNAs are transcribed by RNA polymerase II and undergo maturation through 5′ cap modification, 3′ polyadenylation and splicing. On the other hand, many features of lncRNAs can distinguish them from protein-coding mRNAs. Generally, lncRNAs lack coding potential, expressed at relatively lower levels, prone to localize in the nucleus and evolve faster [7]. Accumulating evidence has supported involvement of a broad spectrum of lncRNAs in a variety of disease states including oncogenesis [8]. In breast cancer research, both oncogenic and tumor suppressive lncRNAs have been identified. They are involved in the regulation of gene expression at various levels that ultimately affect cancer cell growth, apoptosis, migration, invasion and stemness maintenance [9]. Notably, several lncRNAs modulating the TGF-β (transforming growth factor beta) signaling pathway, a central function for mammary development, have been found aberrantly expressed in breast cancer [10, 11].

Linc-ROR (lncRNA ROR) was initially discovered as a modulator for reprogramming of human induced pluripotent stem cells and proven to have strong impact on self-renewal and differentiation of human embryonic stem cells [12, 13]. Since then, dysregulation of linc-ROR has been found in a variety of tumors [14]. In many breast cancer cell lines and tissues, linc-ROR is dramatically upregulated and has been implicated to contribute to malignancy and treatment resistance of advanced breast cancer [15–19]. The oncogenic function of linc-ROR has been linked to the regulation of multiple signaling pathways, which are presumably important for the development and progression of cancers. However, a complete picture of the regulatory network associated with linc-ROR is still missing. This has hampered the application of linc-ROR as a diagnostic biomarker and development of new therapeutic approaches.

We found that linc-ROR was overexpressed in both breast cancer cell lines and patient tissues. The high expression levels of linc-ROR ware associated with poor prognostic outcomes. Further characterization suggested the role of linc-ROR in enhancing proliferation and invasion of breast cancer cells as well as promoting tumor growth in nude mice. We also found that the expression levels of linc-ROR affected several factors in the TGF-β pathway suggesting that the regulatory link between linc-ROR and TGF-β is potentially important for progression of advanced breast cancer. The results provided new insights into the signaling networks associated with the oncogenic action of linc-ROR and might help develop new diagnostic and therapeutic strategies.

## Materials and methods

### Breast tissue samples

Collection of patient samples was approved by the Ethics Committee of Taihe Hospital affiliated to Hubei University of Medicine. The breast tissues were obtained from 94 breast cancer patients who had surgery in the Taihe Hospital between 2015 and 2017. The patients were 31–59 years old (45 ± 13.5 years old on average) without smoking history. Their breast cancer conditions were diagnosed by two pathologists following the American Society of Clinical Oncology guidelines. Both tumor and the adjacent normal tissues were collected. The normal tissues were at least 2 cm away from the edges of the tumors and contained no obvious tumor cells. All the tissue samples were obtained from fresh surgical specimens, flash-frozen in liquid nitrogen, and stored at − 80 °C.

### Cell lines and culture conditions

The human breast cancer cell lines (MDA-MB-231 and MCF-7) and the normal mammary fibroblast cell line (Hs578Bst) were purchased from Beijing Zhongyuan Ltd. and Shanghai Kexing Biotech Ltd. respectively. The MDA-MB-231 cells were maintained in L-15 medium, while the MCF-7 and Hs578Bst cells were maintained in DMEM (Dulbecco’s Modified Eagle’s Medium). All culture media were supplemented with 10% FBS (fetal bovine serum), 100 U/mL of penicillin, and 100 μg/mL of streptomycin, unless otherwise specified. All the cells were incubated at 37 °C in 5% CO_2_.

### Cell transfection

The siRNA specifically targeting ROR (si-ROR) and the scrambled control (si-nc) were synthesized by Shanghai GenePharma Co. Ltd. The RNAs were transfected into cells using the Lipofectamine RNAiMAX reagents (Thermo Fisher Scientific, USA) according to the manufacturer’s protocol. The si-nc and si-ROR sequence are below. si-nc: UUCUC CGAAC GUGUC ACGU; si-ROR: GGAGA GGAAG CCUGA GAGU.

### RNA extraction and qRT-PCR

RNA was isolated from tissues or cells using the TRIzol reagent (Thermo Fisher Scientific) and quantitatively analyzed by qRT-PCR (quantitative reverse transcription polymerase chain reaction). Briefly, RNA was reverse transcribed using the SuperScript First Strand cDNA System kit (Thermo Fisher Scientific) and the DNA was subjected to quantitative PCR in a Thermo Fisher Scientific 7300 Real-Time PCR System. The RNA level of GAPDH (glyceraldehyde-3-phosphate dehydrogenase) was used as an internal control. The delta Ct (threshold cycle) method was used to quantitate gene expression levels, while the delta–delta Ct method was used to determine the relative fold changes in gene expression.

The primers used are listed below. ROR:5′-CTCCAGCTATGCAGACCACTC-3′;5′-GTGACGCCTGACCTGTTGAC-3′. GAPDH:5′-AATGGACAACTGGTCGTGGAC-3′,5′-CCCTCCAGGGGATCTGTTTG-3′.

### Western blot analysis

The harvested cells lysed and the supernatants were quantitated by BCA assays using a Varioskan multimode microplate spectrophotometer (Thermo Scientific, USA). Equal amounts of proteins were subjected to 10% sodium dodecyl sulphate–polyacrylamide gel electrophoresis (SDS-PAGE). The separated proteins were transferred on to nitrocellulose membranes. The membranes were blocked with 10% no-fat milk in phosphate buffered saline (PBS) at 37 °C for 1 h. Specific antibodies against TGF-β (ab31013, abcam), Smad2 (ab40855, abcam), α-SMA (#19245, CST) and GAPDH (ab9485, abcam) were added for overnight incubation at 4 °C. After the antibodies were removed, membranes were incubated with secondary antibodies (ab6721, abcam) for 1 h at 37 °C. Protein bands were detected with the Odyssey Infrared Imaging System (LI-COR Inc., USA). Protein levels were averaged from triplicates.

### MTT assay

Cells were seeded into 96-well plates at a density of 5 × 10^3^/well. The cells transfected with siRNA were incubated for 48 h before being resuspended and seeded into the 96-well plates. The MTT solution (20 μL) was added to the plates at 12-, 24-, 48-, and 72-h time points and the cells were further incubated for 4 h at 37 °C. The culture medium was then replaced by 150 μL DMSO (dimethyl sulfoxide) and the cells were oscillated for 15 min. The absorbance at 490 nm was determined with an enzyme-labeled analyzer.

### Cell invasion assays

Cell invasion was analyzed using Transwell invasion chambers (Corning, USA). Briefly, 3  ×  10^4^ transfected cells in serum-free DMEM were added onto the upper chambers of 8 μm-diameter Transwell inserts pre-coated with Matrigel (R&D Systems, USA), and 0.5 mL DMEM with 10% FBS was added to the lower chamber. After 48 h incubation at 37  °C, cells on the bottom chamber were fixed with 70% ethanol, stained with 0.1% crystal violet, and photographed under an inverted microscope. The numbers of cells in five random fields per well were counted and averaged as the invading cell number.

### Tumor xenograft model

The MCF-7 and MDA-MB-231 breast cancer cells were transfected with lenti-control (sh-nc) or lenti-shRNA-ROR (sh-ROR) (GeneChem, Shanghai, China) and selected for puromycin resistance (10 μg/mL). Female nude mice, 4–5 weeks of age, were purchased from Vital River Laboratory Animal Technology Ltd. (China). Stable MCF-7 and MDA-MB-231 cells (2 × 10^6^ cells in 50% matrigel, BD Biosciences, USA) with or without shRNA transfection were subcutaneously injected into the right flank of nude mice. The formation and growth of tumor volume in the nude mice were monitored. At day 31, the mice were sacrificed and the tumors were isolated and weighted.

Tumor volume was calculated by the formula below.

$${\text{Volume}}\, = \,{\text{Width}}^{ 2} \, \times \,{{\text{Length}} \mathord{\left/ {\vphantom {{\text{Length}} 2}} \right. \kern-0pt} 2}$$

The primer sequences for sh-nc and sh-ROR are listed below.

GATCCCCTTCTCCGAACGTGTCACGTTTCAAGAGAACGTGACACGTTCGGAGAATTTTTC.

GATCCCCCCTGAGAGTTGGCATGAATTTCAAGAGAATTCATGCCAACTCTCAGGTTTTTC.

### Statistical analysis

All statistical analyses were carried out using the SPSS 17.0 software (SPSS Inc., USA). Most datasets were analyzed using Independent Samples t Test. The overall survival was estimated using the Kaplan–Meier method. The results with *p *< 0.05 were considered statistically significant.

## Results

### High expression of linc-ROR is linked to breast cancer

**Fig. 1:**
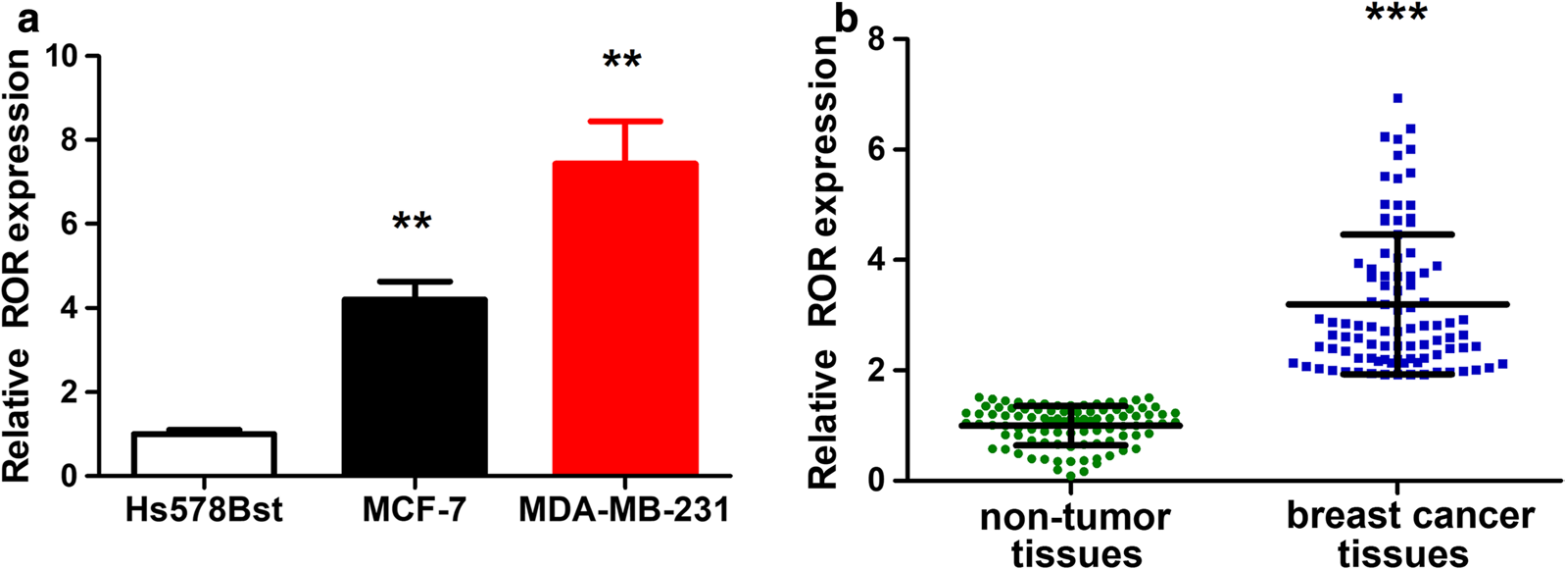
Upregulation of linc-ROR in breast cancer cell lines and tumor tissues. **a** Bar plot comparing the linc-ROR RNA levels in different cell lines. The MCF-7 and MDA-MB-231 cells are breast cancer cells, while the Hs578Bst cells are normal mammary fibroblast cells. The linc-ROR RNA levels were quantitated by qRT-PCR. GAPDH RNA was used as an internal control. Data were averaged from triplicate experiments. **b** Statistical comparison of linc-ROR expression levels in patient tissue samples. Linc-ROR RNA levels were quantitated from breast tumor tissues and the adjacent normal tissues using the same method described above. **p < 0.01, ***p < 0.001, vs the control

Dysregulation of linc-ROR has been found in a variety of tumors including breast cancer. We compared the expression levels of linc-ROR in cells cultured in vitro and found that the expression in two breast cancer cell lines (MCF-7 and MDA-MB-231) were higher than that in the normal mammary fibroblast cell line (Hs578Bst) (Fig. 1a). In order to find clinical relevance, we collected tumor tissues and the adjacent normal tissues from 94 patients and statistically compared the expression levels of linc-ROR between these two groups (Fig. 1b). Indeed, linc-ROR was upregulated in the tumor tissues. The results suggested that high expression of linc-ROR might be important in breast cancer.

**Fig. 2:**
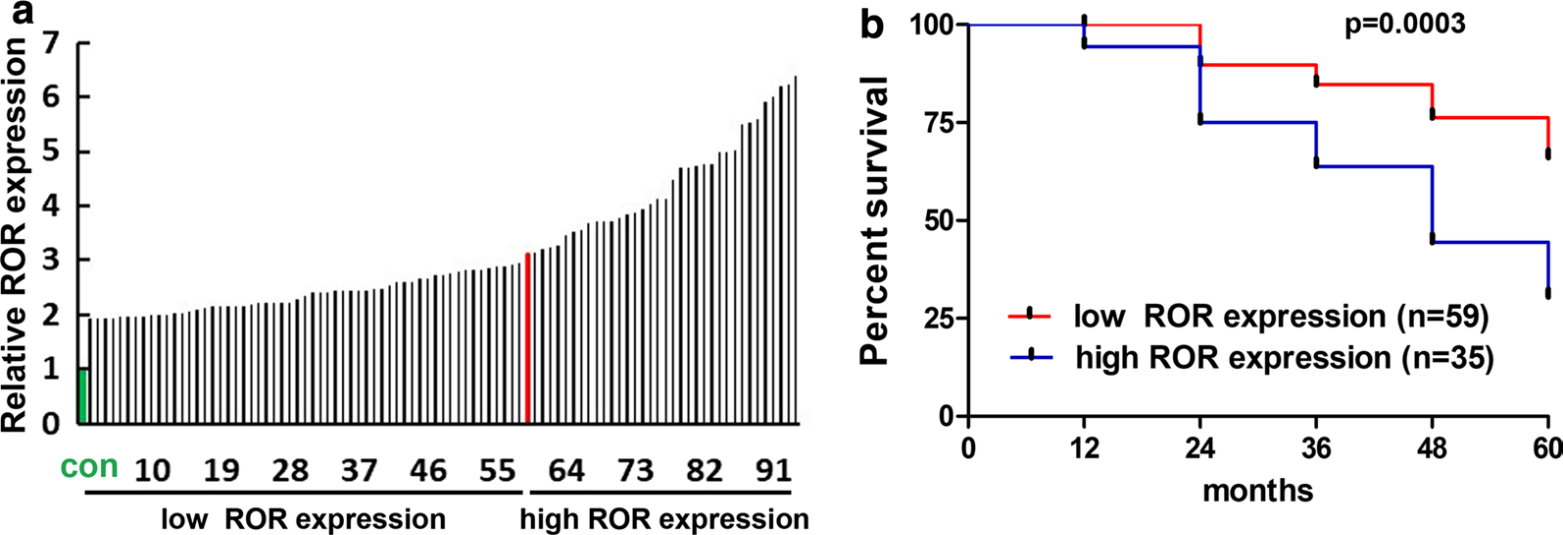
Comparison of prognostic survival rates between Linc-ROR low- and high-expression groups. **a** Schematic presentation of two patient groups with low- and high-expression levels of linc-ROR. The expression levels of all 94 patients (black bars) were ordered from low to high. The control expression level (green bar) was normalized to 1. The demarcation was set to the patient (red bar) with 3.2-fold increase in the level of linc-ROR. **b** Kaplan–Meier overall survival curves of the low- and high-expression groups. Breast cancer patients with high expression of linc-ROR showed a poorer prognosis than those with low expression

We tried to further assess the expression of linc-ROR in clinical prognosis of breast cancer patients. We first divided the 94 patients into two groups, the low- and high-expression groups, according to the linc-ROR levels in the tumor tissues. The demarcation was set at a patient with 3.2-fold increase in linc-ROR expression (Fig. 2a). When the 60-month survival fractions were compared, the high-expression groups showed faster decline in survival (Fig. 2b). This reinforced our hypothesis that linc-ROR may be an oncogenic factor for the development of breast cancer.

### Linc-ROR is required for the enhanced proliferation and invasion of breast cancer cells

**Fig. 3:**
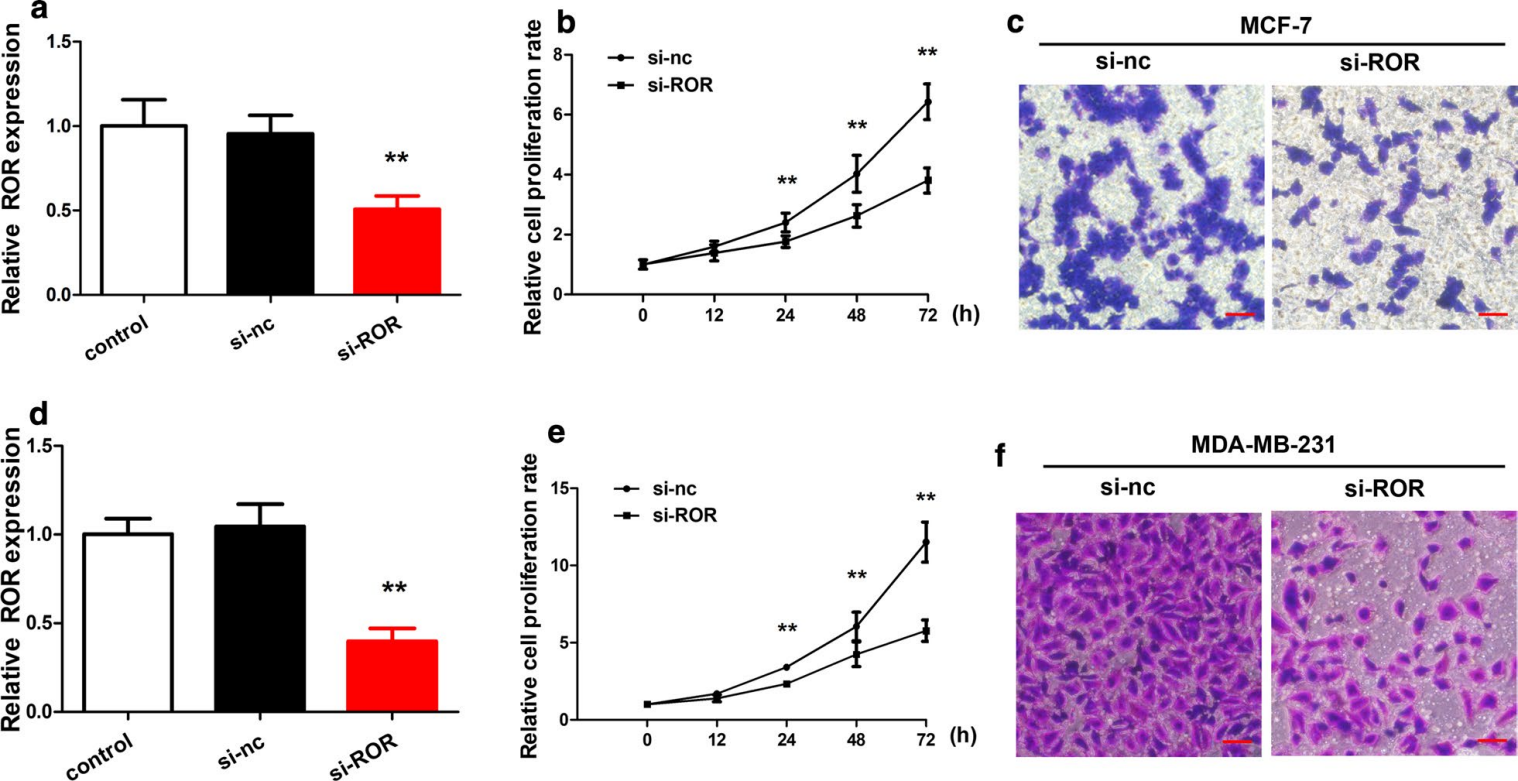
Linc-ROR knockdown reduced cell proliferation and invasion. **a** Comparison of the linc-ROR levels in the cultured MCF-7 breast cancer cells with or without linc-ROR knockdown. Linc-ROR was knocked down by the si-ROR interference RNA. Cells were also untransfected (control) or transfected with a scrambled negative-control siRNA (si-nc) for comparison. **b** Time courses of MCF-7 proliferation. Cell proliferation rates were compared between the cells transfected with si-ROR or si-nc. The proliferation rates were quantitated every 12 h over 3 days. Linc-ROR knockdown by si-ROR led to slower proliferation. **c** Comparison of MCF-7 cell invasion. The cells transfected with si-ROR or si-nc were assessed in transwell assays. The scale bars are 100 μm. The data points were averaged from triplicates. **p < 0.01 vs the control. **d**–**f** Comparison of proliferation and invasion for the MDA-MB-231 breast cancer cells transfected with si-ROR or si-nc. The panels are arranged as **a**–**c**

We examined the importance of linc-ROR in the MCF-7 and MDA-MB-231 breast cancer cells by analyzing the proliferation and invasion of those cells in vitro. When the level of linc-ROR was reduced by introduction of interference RNA targeting the linc-ROR transcripts (si-ROR) in both types of cancer cells, the proliferation and invasion of those cells were partially inhibited, supporting the potential importance of linc-ROR for the tumorigenic phenotypes of the cancer cells (Fig. 3).

### Knocking down of linc-ROR renders tumor growth

**Fig. 4:**
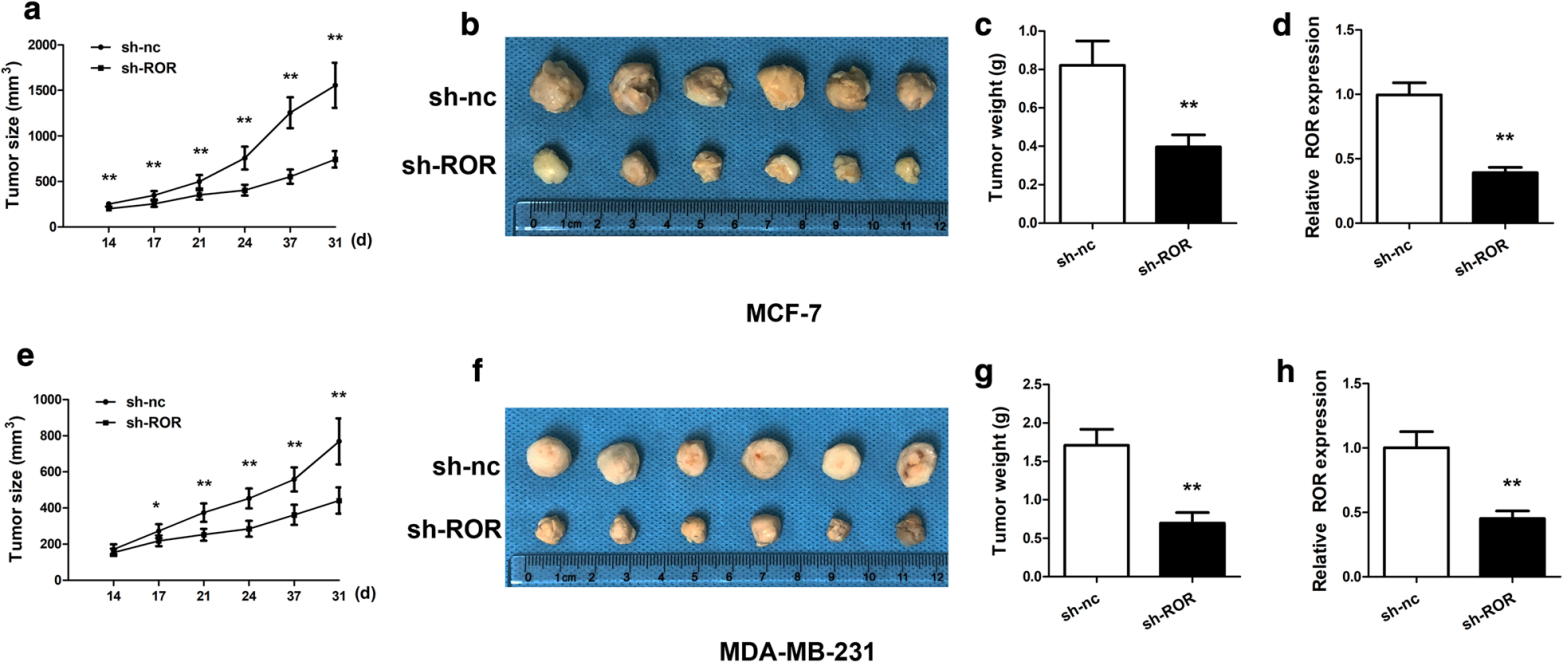
Linc-ROR knockdown reduced tumor growth in nude mice. **a** Time-course comparison of tumor growth from the MCF-7 cells. The cells were transfected with si-ROR or si-nc and injected into nude mice. After 14 days, tumor sizes were measured twice a week over 3 weeks. **b** Image of tumors harvested at day 31. **c** Comparison of averaged tumor weights. The tumors grown from the MCF-7 cells transfected with si-ROR were smaller. **d** Relative ROR expression of tumors was confirmed. The expression of ROR of tumors from the MCF cells transfected with si-ROR was inhibited. **e–h** Comparison of tumors derived from the MDA-MB-231 cells, which were transfected with si-ROR or si-nc and injected into nude mice. The panels are arranged as **a**–**c**. **p < 0.01 vs the control

We also injected both types of cancer cells into nude mice and compared the growth of tumors in the animals. When si-ROR were transfected into the cancer cells to suppress the expression of linc-ROR, the growth of the tumors formed from the injected cells was apparently slower during the 1-month measurement period. After the tumors were isolated from the terminated animals, the weights of the tumors were also compared. Indeed, knocking down linc-ROR in both types of cancer cells rendered formation of smaller tumors (Fig. 4). The results suggested that the linc-ROR is potentially critical for the development of breast cancer.

### Linc-ROR knockdown inhibits the activation of the TGF-β signaling pathway

**Fig. 5:**
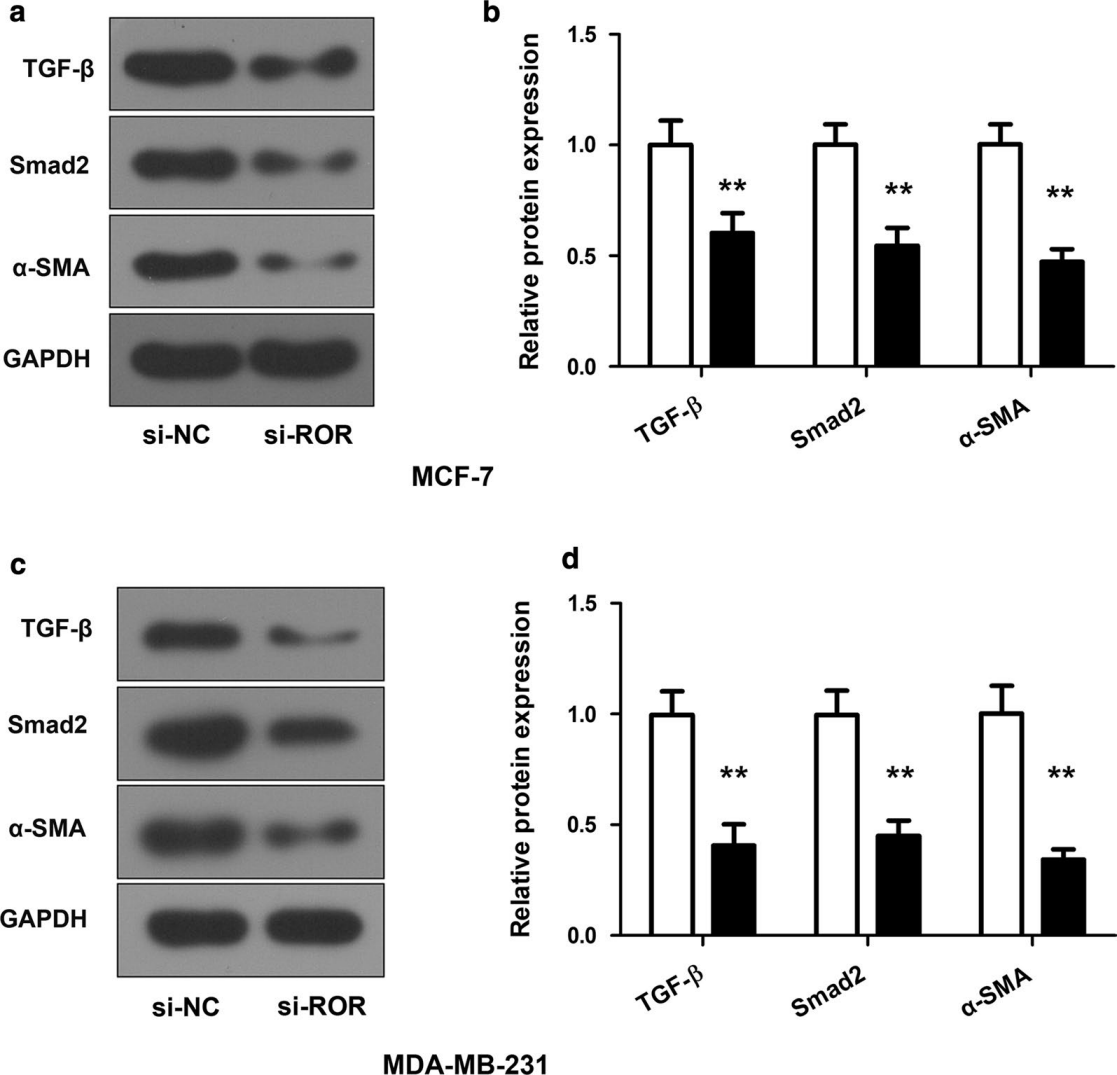
Linc-ROR modulates the TGF-β signaling pathway. **a** Western blot probing TGF-β, Smad2 and α-SMA in the MCF-7 cells. The cells were transfected with si-ROR or si-nc. GAPDH was blotted as an internal reference. **b** Quantitative comparison of the expression levels of TGF-β, Smad2 and α-SMA. The protein expression levels in the MCF-7 cells transfected with si-nc were normalized to 1. The cells transfected with si-ROR showed reduced expression of all three proteins. **c**, **d** Comparison of the protein expression levels of TGF-β, Smad2 and α-SMA in the MDA-MB-231 cells. The panels were arranged as **a**, **b**. Data were Mean ± SD derived from three independent experiments. *p < 0.05 vs the control

In order to understand how linc-ROR may support the development of breast cancer, we analyzed its impact on TGF-β, a critical signaling factor orchestrating mammary epithelial development and contributing to cancer progression in the advanced stages. When linc-ROR was knocked down in the MCF-7 and MDA-MB-231 breast cancer cells, the expression levels of TGF-β were also diminished. As a result, the downstream factors, such as Smad2 and α-SMA, were also downregulated (Fig. 5). These results indicated that linc-ROR likely modulates the TGF-β signaling pathway to trigger the expression of a series of factors involved in the progression of breast cancer.

## Discussion

Our study has confirmed aberrant overexpression of linc-ROR in both breast cancer cell lines and patient tumors, demonstrating its strong correlation with poor prognostic outcomes. The results, together with those from many previous studies, have thus reinforced the hypothesis that this lncRNA is an oncogenic factor that can promote the development and progression of breast cancer [15–19]. Our results have further suggested that the TGF-β signaling pathway may be a downstream target of linc-ROR, responsible for promoting breast cancer. This is line with the previous conclusion that TGF-β is a central signaling molecule in mammary development as well as tumorigenesis [10].

Many studies have recognized that the function of TGFβ in the progression of breast cancer can be different depending on the stage of cancer [10, 20, 21]. In normal conditions, TGF-β has been shown to inhibit cell cycle and promote apoptosis that together significantly contribute to the suppressive role in the initiation and progression of tumorigenesis [22]. In the late stages of tumor progression, however, TGF-β acts as a tumor promoter largely by inducing and promoting epithelial-to-mesenchymal transitions [23, 24]. Our study has indicated that overexpression of linc-ROR is required to constitutively upregulate critical factors in the TGF-β signaling pathway. This suggests that the oncogenic activity of linc-ROR is at least needed for the progression of breast cancer in the advanced stages, although it likely regulates a highly complex signaling network that can impact many other biological processes that ultimately contribute to multiple steps along the progression of the disease.

Although the survival rates have been gradually increased in some countries because of remarkable improvements in the understanding and management of breast cancer, the disease is still a major medical burden worldwide [25]. Major challenges include developing cost-effective diagnosis, treating metastatic cancer and overcoming drug resistance in targeted therapies.

## Conclusion

Our data suggest that linc-ROR, in combination with other lncRNAs associated with breast cancer, may serve as a clinically beneficial biomarker for detecting neoplastic cells, differentiating the different stages of breast cancer and predicting prognostic outcomes. Furthermore, blocking the functional impact of linc-ROR may represent a novel therapeutic strategy that may help overcome a plethora of cellular mechanisms exploited to favor drug resistance.
